# Supplementary material for: Bioreactor-based mass production of human iPSC-derived macrophages enables immunotherapies against bacterial airway infections
Source: Nat Commun. 2018 Nov 30;9:5088. doi: 10.1038/s41467-018-07570-7 (PMC6269475; doi:10.1038/s41467-018-07570-7)
Supplement: Supplementary file 1 — Supplementary Information [file 41467_2018_7570_MOESM1_ESM.pdf]

Supplementary Information

Bioreactor-based mass production of human iPSC-derived macrophages enables immunotherapies against bacterial airway infections

Ackermann and Kempf et al.

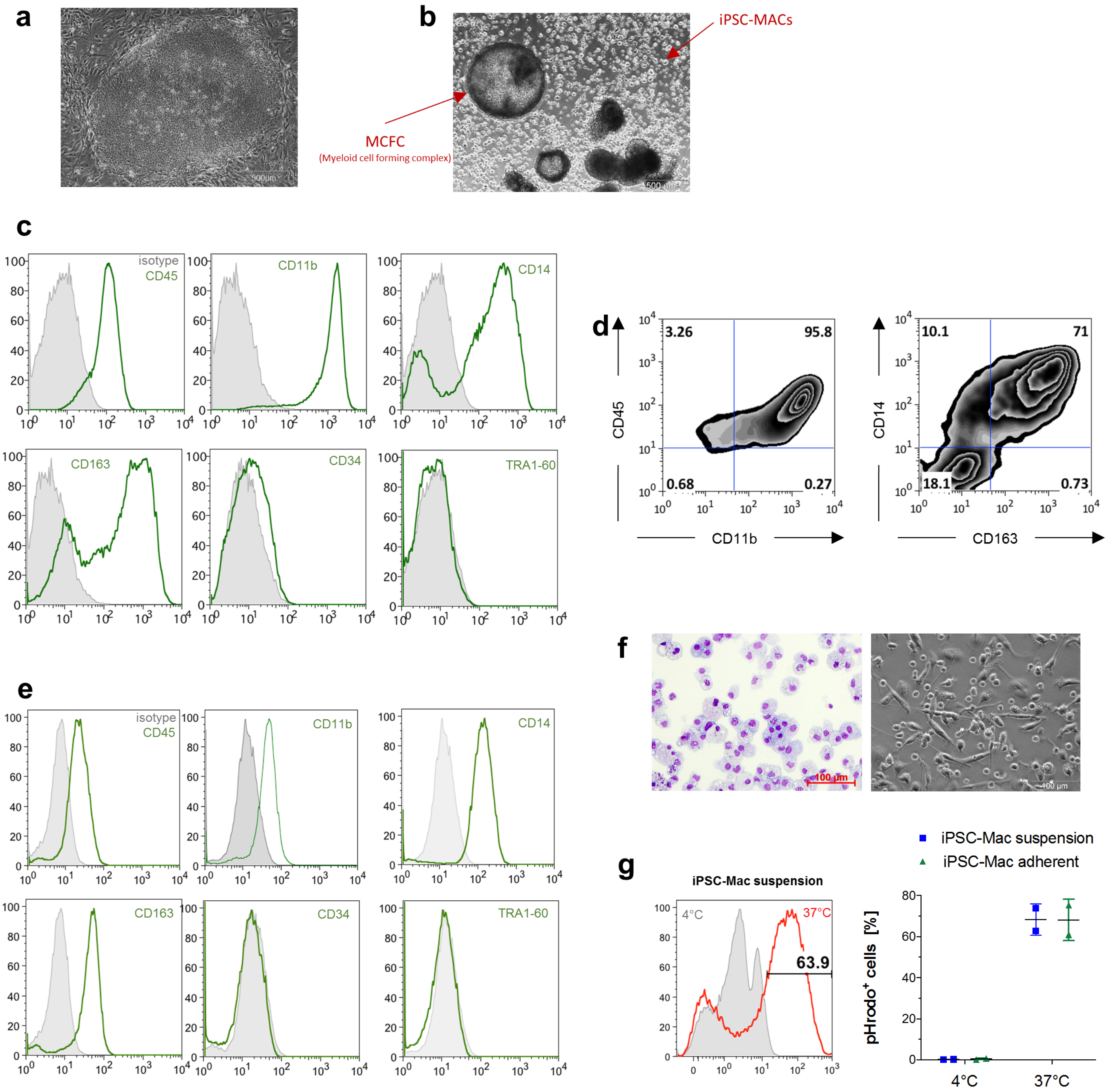

Supplementary Figure 1. Hematopoietic differentiation of human iPSC in suspension culture. (a) Representative brightfield image of undifferentiated human iPSC (CD34iPSC16). Scale bar 500µm. **(b)** Representative brightfield image of CD34iPSC16 differentiated towards a myeloid cell forming complex (MCFC) producing iPSC-derived macrophages (iPSC-Mac). Scale bar 500µm. **(c and d)** Flow cytometry of freshly harvested iPSC-Macs (pre-gated on viable cells in forward and sidewards scatter (FSC/SSC) analysis, gray filled: respective isotype control, green line: surface marker). **(e)** Flow cytometry of iPSC-Mac terminally differentiated by M-CSF for a minimum of 7 days (pre-gated on viable cells in forward and sidewards scatter (FSC/SSC) analysis, gray filled: respective isotype control, green line: surface marker). **(f)** Cytospin staining (left) and brightfield microscopy (right) of terminally differentiated iPSC-Mac. Scale bars 100µm. **(g)** Phagocytosis of pHrodo-labeled *E. coli* particles by iPSC-Mac at 4°C (negative control) or at 37°C. Representative plot for suspension-cultured derived iPSC-Mac (left) and quantification of n=2 (right). Human iPSC-derived macrophages from adherent based differentiation cultures (green) were used as controls (n=2 individual experiments, mean±SD).

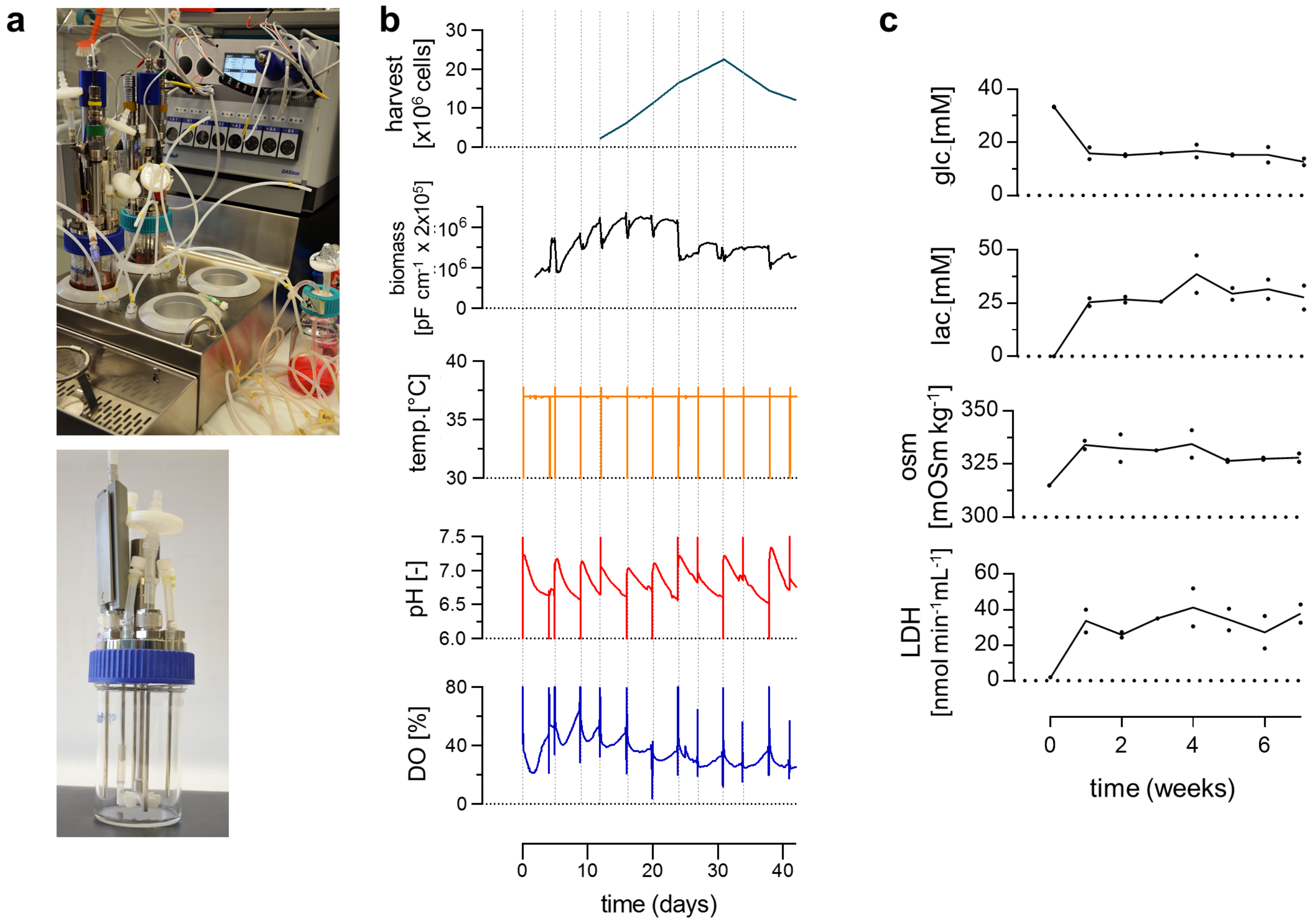

Supplementary Figure 2. Continuous hematopoietic differentiation using stirred tank bioreactors. **(a)** Pictures of DASBox Mini Bioreactor system (upper picture) and respective empty bioreactor and the 8-blade impeller (lower picture) used in this study. **(b)** Continuous monitoring of several parameters (cell harvest, biomass, temperature, pH and oxygen) during macrophage generation for a representative bioreactor run. **(c)** Analysis of different factors in the bioreactor supernatant (glc: glucose, lac: lactate, osm: osmolarity, LDH: lactate dehydrogenase; data from two biological independent bioreactor runs (dots) and respective mean (line).

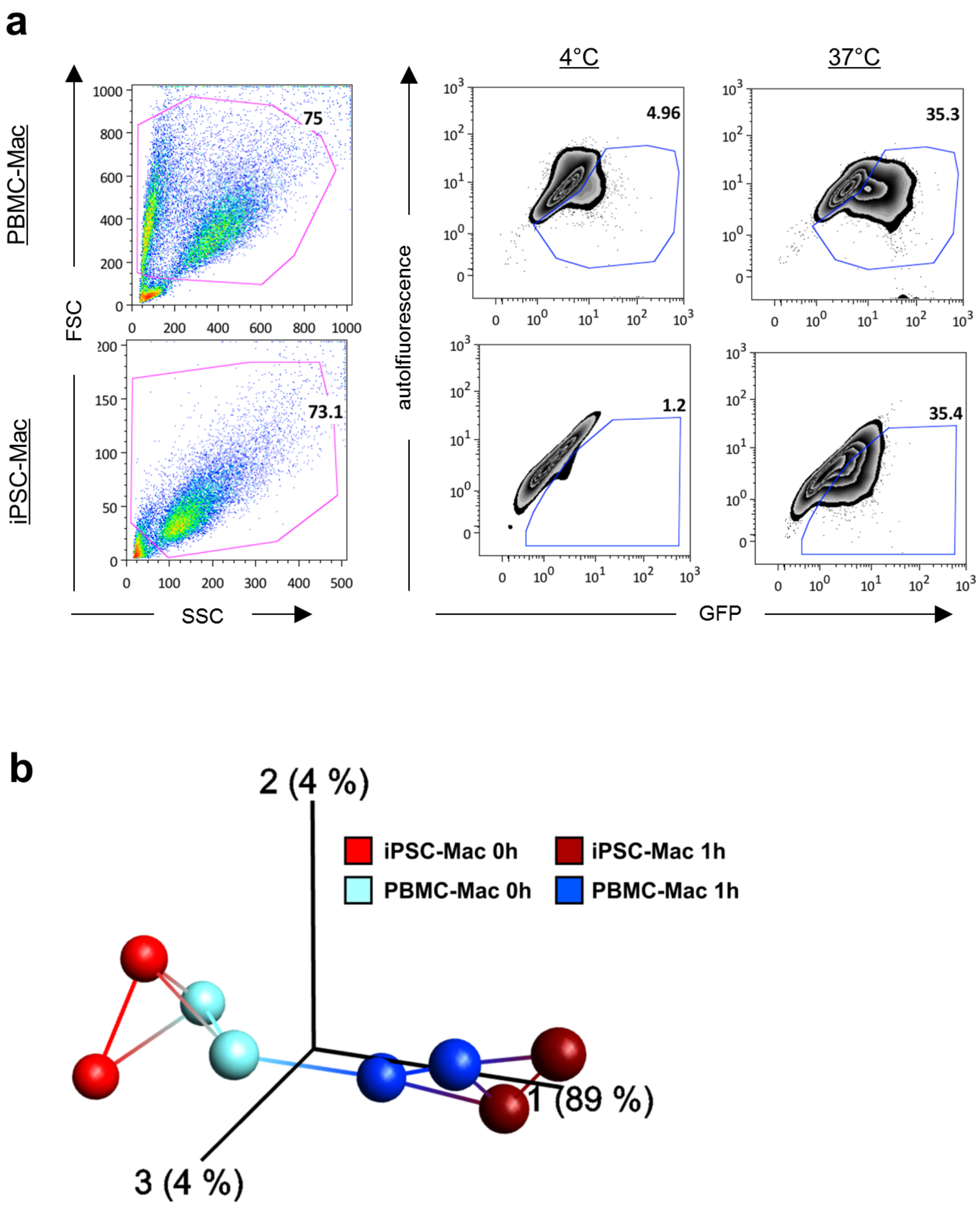

Supplementary Figure 3. Response of iPSC-Mac and PBMC-Mac to pathogen exposure. (a) Phagocytic activity of terminally differentiated iPSC-Mac and PBMC-Mac 2h after incubation with GFP-labeled *P. aeruginosa* (PAO1) at 4°C or 37°C analyzed by flow cytometry. Representative results for iPSC-Mac and PBMC-Mac. **(b)** Principal component analysis of differentially expressed inflammatory genes (GO:0006954; $p<0.05$) in terminally differentiated iPSC-Mac and PBMC-Mac 1h post exposure to *P. aeruginosa* (PAO1) and respective controls (0h).

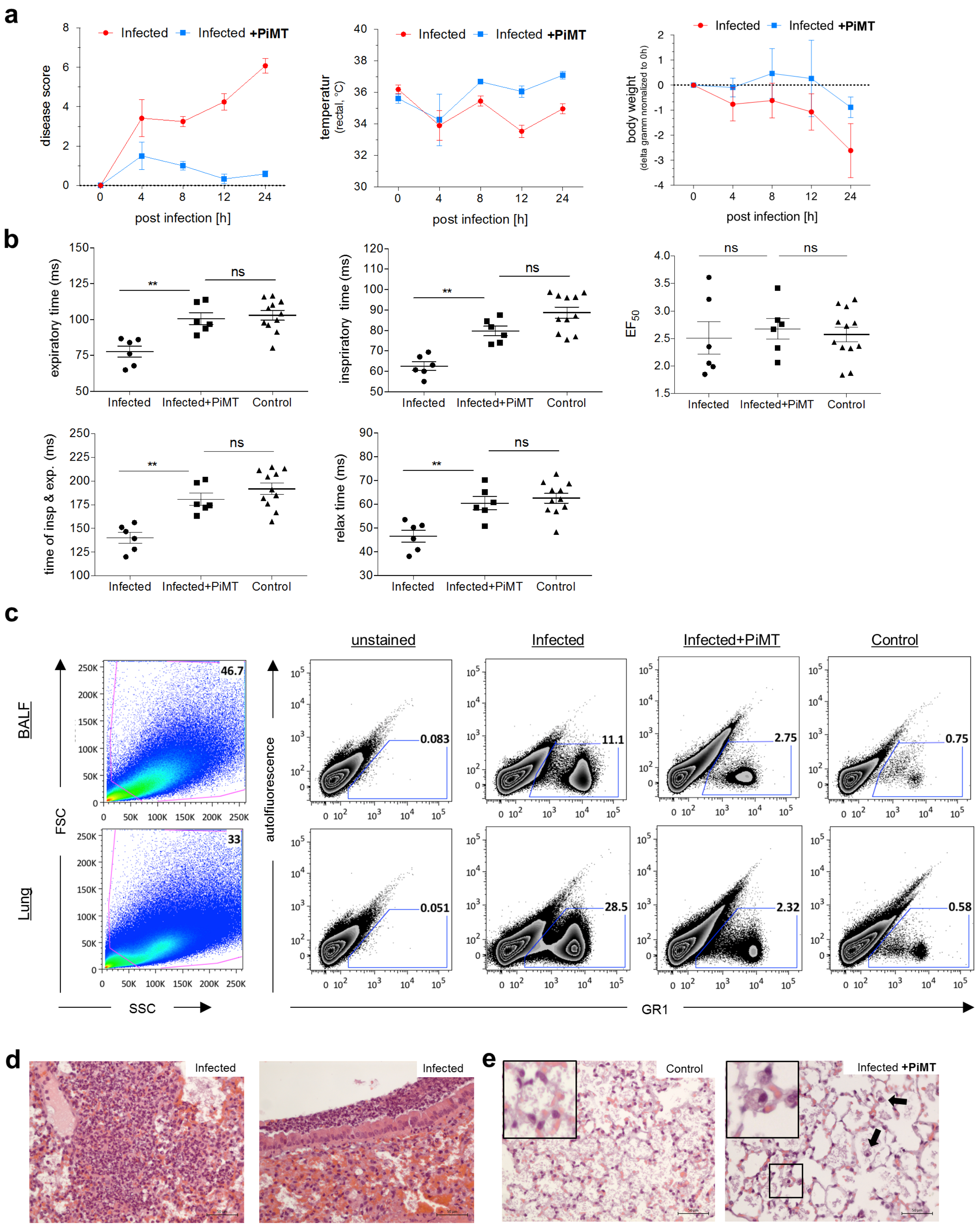

Supplementary Figure 4. Pulmonary infection and simultaneous macrophage transplantation in hIL-3/GM-CSF KI mice. (a) Time courses of disease score, temperature and body weight (normalized to the respective weight before infection) of hIL-3/GM-CSF KI mice from infected or infected+PiMT groups (n=6 animals/group, mean±s.e.m). **(b)** Lung function (expiratory time, inspiratory time, time of insp.&expr., relax time and EF_{50}) in hIL-3/GM-CSF KI animals measured by head-out bodyplethysmography 24h post-infection. Values measured before infection served as control values (n=6 animals/group, mean±s.e.m). **(c)** Flow cytometry analysis of BALF and lung. Percentages of mouse granulocytes (determined as $GR1^{+}$ cells among viable cells) in BALF and Lung. Gating strategy and representative results for one animal per group. **(d,e)** Pathohistology of right murine lungs showing **(d)** massive granulocyte infiltration in infected hIL-3/GM-CSF KI animals and **(e)** macrophages in infected+PiMT animals only (representative image, n=3). Scale bar, 50µm. Control: non-transplanted animal (**p<0.01, ns denotes not significant; statistical significances were assed using one-way ANOVA with Dunnett's multiple comparisons test).

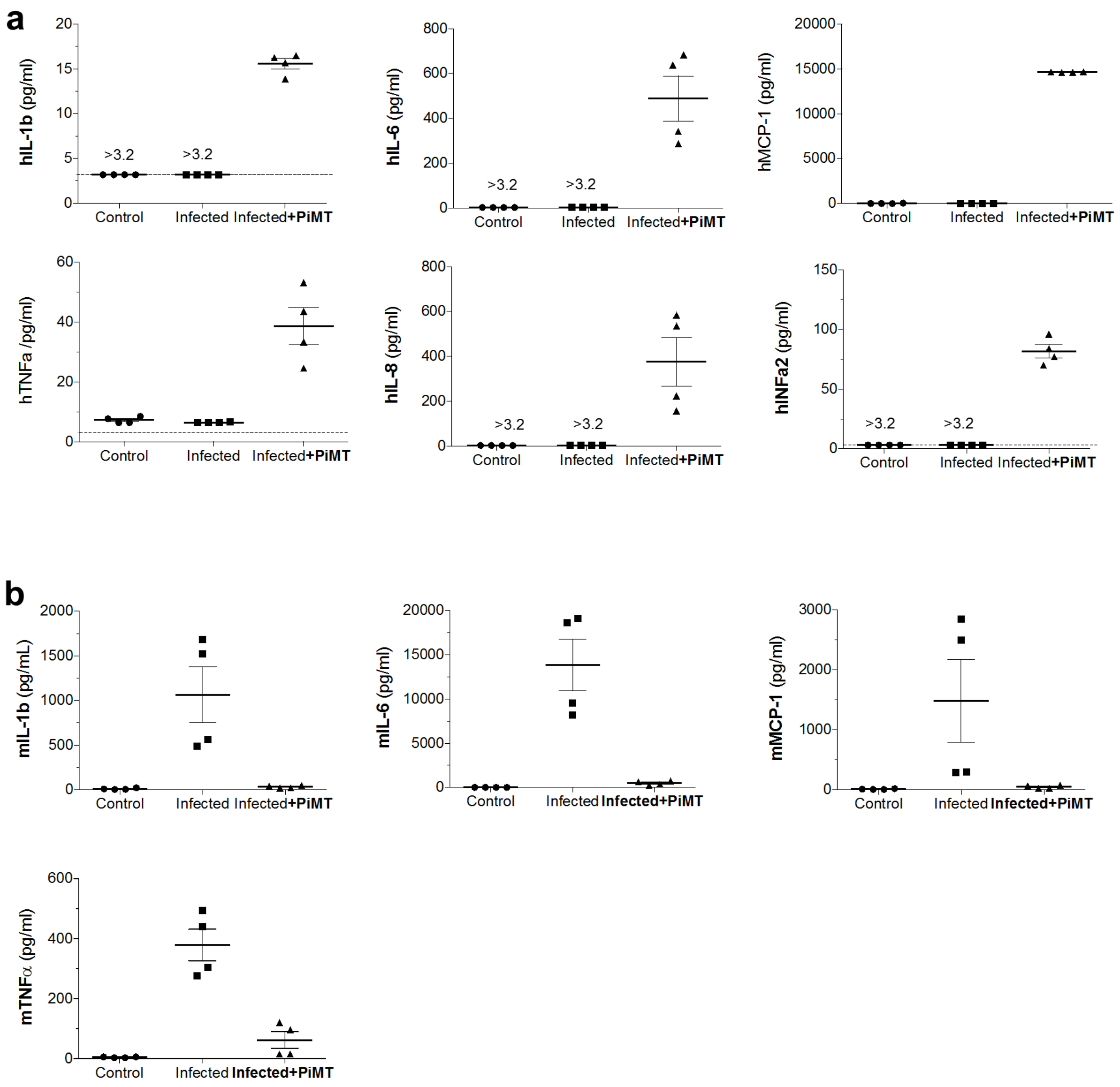

Supplementary Figure 5. Pulmonary infection and therapeutic transplantation of iPSC-Mac in hIL-3/GM-CSF KI mice. Levels of **(a)** human and **(b)** murine cytokines in BALF of healthy control, infected only and infected+PiMT treated animals (n=4: 2 animals and 2 technical duplicates, mean±s.e.m, dotted line indicate detection limits).
