# Supplementary material for: Bioreactor-based mass production of human iPSC-derived macrophages enables immunotherapies against bacterial airway infections
Source: Nat Commun. 2018 Nov 30;9:5088. doi: 10.1038/s41467-018-07570-7 (PMC6269475; doi:10.1038/s41467-018-07570-7)
Supplement: Supplementary file 3 — Description of Additional Supplementary Files [file 41467_2018_7570_MOESM3_ESM.pdf]

Description of additional Supplementary Information

Supplementary Movie 1. Stirred tank bioreactor continuously generating iPSC-Mac from MCFC in suspension.
